# Barriers and enablers to deprescribing in long-term care: A qualitative investigation into the opinions of healthcare professionals in Ireland

**DOI:** 10.1371/journal.pone.0274552

**Published:** 2022-12-15

**Authors:** Clara H. Heinrich, Sheena McHugh, Suzanne McCarthy, Maria D. Donovan

**Affiliations:** 1 School of Pharmacy, University College Cork, Cork City, Co. Cork, Ireland; 2 School of Public Health, University College Cork, Cork City, Co. Cork, Ireland; Universita degli Studi di Milano, ITALY

## Abstract

**Introduction:**

The prevalence of polypharmacy increases with age, increasing the exposure of older adults to potentially inappropriate medications (PIMs). Deprescribing has been shown to reduce PIMs for older residents in long-term care; however, deprescribing is not universally implemented. This study aims to identify the barriers and enablers to deprescribing in Irish long-term care facilities from the healthcare professionals’ (HCPs) perspective.

**Methods:**

A qualitative descriptive approach was conducted using semi-structured interviews with HCPs working in long-term care (general practitioners, pharmacists and nurses). Purposive sampling with maximum variation was applied to select long-term care sites to identify HCPs, supplemented with convenience sampling of post-graduate HCPs from University College Cork. Data was thematically analysed and mapped to a framework of deprescribing barriers and enablers informed by the Theoretical Domains Framework.

**Results:**

Twenty-six HCPs participated from 13 long-term care facilities. The main barriers and enablers identified mapped to five domains. Barriers included insufficient resources, lack of co-ordination between healthcare settings and negative social influences. Additional barriers exist in private settings including deprescribing awareness, commitment and the need for incentives. Deprescribing enablers included interprofessional support and patient social influence. To encourage deprescribing, potential enablers include HCP education, pharmacist role expansion and tailored deprescribing guidelines within a structured process.

**Conclusion:**

Interventions to support deprescribing should build on existing systems, involve stakeholders and utilise guidelines within a structured process. Any intervention must account for the nuanced barriers and enablers which exist in both public and private settings.

## Introduction

The prevalence of polypharmacy, generally considered the use of five or more medications, is rising [1–3]. Globally, polypharmacy increases with age, which is mirrored in Ireland, with 31% of the population over 65 taking five or more medications, rising to 37% in patients over 75 [4,5]. While polypharmacy can be appropriate when all medications are clinically indicated, inappropriate polypharmacy is of concern [6–8]. Age-related physiological changes can cause older adults to be exposed to adverse consequences from medications which were once indicated and appropriate [9–11]. Polypharmacy has been associated with potentially inappropriate medications (PIMs) which are linked with negative outcomes for older adults, such as adverse drug reactions, hospitalisations and mortality, particularly for frail older adults resident in long-term care [12–18].

Internationally, PIM use is highly prevalent among older adults resident in long-term care [19]. A Belgian study identified that 64% of frail long-term care patients had one or more PIM [20]. Similarly, this has been identified as a significant problem in Ireland, with studies documenting that 70% of patients were prescribed at least one PIM [14,21]. Considering the relationship between polypharmacy, PIMs and adverse drug reactions for long-term care patients, efforts are required to improve the appropriateness of prescribing in this setting.

Deprescribing, defined as a patient-centred process of medication withdrawal, is an effective intervention to tackle inappropriate polypharmacy as it aims to improve health outcomes through discontinuing medications that are either potentially harmful or no longer required [22,23]. A systematic review of deprescribing interventions in long-term care found evidence of a 59% reduction in PIMs, improved patient safety and an increase in medication appropriateness [24]. Long-term care-specific deprescribing benefits include improved medication adherence, quality of life and reduced time spent on medication administration [25].

Considering the high incidence of PIMs among frail older adults, deprescribing requires further attention. Despite best-practice guidelines, a US study documented limited PIM discontinuation rates, for older adults with limited life expectancy [26]. Similarly, deprescribing interventions, such as medication reviews, are considered unlikely to be incorporated into daily practice [27]. Researchers have called for an increased focus on implementation science to bridge the “research-to-practice gap”, which may increase deprescribing engagement in clinical practice by, for example, tailoring interventions to the long-term care environment [28,29]. As identified by the Medical Research Council’s framework for developing and evaluating complex interventions, context and stakeholder engagement are core elements to intervention design [30]. To develop a successful deprescribing intervention, a better understanding of HCPs’ deprescribing behaviour and the barriers and enablers which they experience in Irish long-term care settings is required, as these are context-specific [31].

In Ireland, different organisational models for long-term care exist. Long-term care facilities are divided into state-funded (public) and private-owned (private) sites, with 80% of settings owned and operated by private providers [32]. Bolmsjö *et al* compared studies, investigating the deprescribing opinions of long-term care General Practitioners (GPs), in two different countries; Australia, which has both public and private funding, like the Irish model, and Sweden, where all long-term care is state funded. Results indicated contrasting opinions about interest in long-term care work and the need for financial reimbursement [33]. Understanding the barriers and facilitators in both organisational models is imperative to develop an intervention that fits with and addresses the challenges in a specific healthcare context.

The primary aim of this study is to generate an understanding of barriers and enablers to deprescribing in Irish long-term care facilities, which are perceived to exist by HCPs working in and with that setting. This will provide key considerations when designing an intervention to increase deprescribing. A secondary aim is to compare barriers and enablers experienced by HCPs in public and private settings, due to the key differences in funding and governance between the settings.

## Materials and methods

### Design

Qualitative description was the chosen methodology for this study [34]. Data was collected using semi-structured interviews. Ethical approval was granted for this study from University College Cork’s Social Research Ethics Committee Log 2021–068.

### Study participants

Purposive sampling strategies were used to sample sites for this study. Initially, stratified sampling was used, with the sampling criterion being public/private status. Sites were selected from a stratified list of long-term care facilities in the south/southwest (Cork/Kerry) region of Ireland (n = 98) [35]. From these settings, eligible participants were HCPs involved in the direct care of long-term care residents, including GPs, pharmacists and nurses. Consultants were not invited as GPs are primarily responsible for the medical needs of residents in Irish long-term care facilities [36]. Healthcare assistants were not included as they are not involved in medical decisions [37]. Long-term care facilities were contacted via phone call and/or email to introduce the project, along with a request to advertise the project to their HCPs. Follow up emails were sent within three weeks of initial contact. Management staff at the long-term care facilities referred interested nurses and HCPs working off site (GPs and pharmacists), to the researcher. In addition, to increase the sampling pool, a convenience sample of HCPs working in long-term care, were recruited through postgraduate courses in University College Cork. An email was distributed by university staff to post-graduate students and academics introducing the project, recruiting HCPs who worked in long-term care.

Information power was used to determine an appropriate sample size. By applying the five established criteria of information power, an initial sample of between 6–10 participants per HCP group, divided equally between public and private long-term care facilities, was deemed sufficient [38].

### Data collection

One-to-one semi-structured interviews were conducted via Microsoft Teams with HCPs. Written, informed consent was obtained from all participants. An interview guide was developed and tailored for each HCP group, guided by the TDF-informed framework of deprescribing barriers and enablers in long-term care facilities [39]. Each domain of the framework informed a question in the topic guide. Additional general questions were included, allowing participants to discuss any factor which they felt was important for deprescribing in long-term care. The guide was reviewed by members of the research team who had experience in qualitative research and refined based on feedback. The interview guide was piloted with a pharmacist, GP, and nurse, all with previous experience of working in long-term care. This confirmed that the guide was clear, understandable and relevant to each HCP group and resulted in no change to the topic guide. As the focus of the study was not related to demographic factors, no demographic details were collected.

All interviews were audio or video-recorded, with the consent of the HCP. Following each interview, recordings were replayed to facilitate familiarisation. All interviews were transcribed verbatim, anonymised and imported into QSR NVivo Version 12® to facilitate analysis.

### Data analysis

The analytical strategy involved two processes drawing on the principles of thematic analysis [40]. In the first stage of analysis, interviews were analysed inductively by the primary researcher to develop themes from the data. This stage drew on the principles of Braun and Clarke’s reflexive thematic analysis including familiarisation, generating initial codes, identifying initial themes, reviewing, and developing themes related to deprescribing barriers and enablers [40]. Inductive analysis can support the theory driven deductive approach and offers both a conceptually and contextually rich understanding of the barriers and enablers to deprescribing [41].

This was preferred over direct mapping to the TDF to allow for inclusion of non-TDF related material.

In the second stage, themes were mapped to domains of the deprescribing framework [39]. A second researcher reviewed the inductive themes, with supporting quotes and independently mapped these to the deprescribing framework. Any discrepancies were resolved during regular meetings with the research team, to discuss inductive codes, mapping, and dominant patterns.

Finally, domains of the deprescribing framework were compared according to professional role and type of facility (public/private) to investigate differences. This was conducted independently by two researchers. Meetings were organised to discuss and agree on findings.

To prioritise potential targets for a deprescribing intervention, dominant domains and constructs were identified, based on two criteria (i) frequency with which the barrier/enabler was mentioned and (ii) the importance attached to it by participants.

The consolidated criteria for reporting qualitative research (COREQ-32) statement was used to inform reporting of the findings (Appendix 1). Transcripts were not returned nor was participant checking performed.

## Results

A total of 26 interviews of HCPs from 13 long-term care facilities were conducted between July and October 2021, as identified in Table 1. Twenty participants were recruited from long-term care settings and six through academic networks. The interviews had a mean length of 24 mins (range 9 mins-65 mins).

**Table 1:** Characteristics of interview participants.

Long-term care settings (n = 13)	Healthcare professionals (n = 26)		
	GP (n = 7)	Pharmacist (n = 7)	Nurse (n = 12)
Public (n = 4)	4	3	6
Private (n = 9)	3	4	6

**Fig 1:**
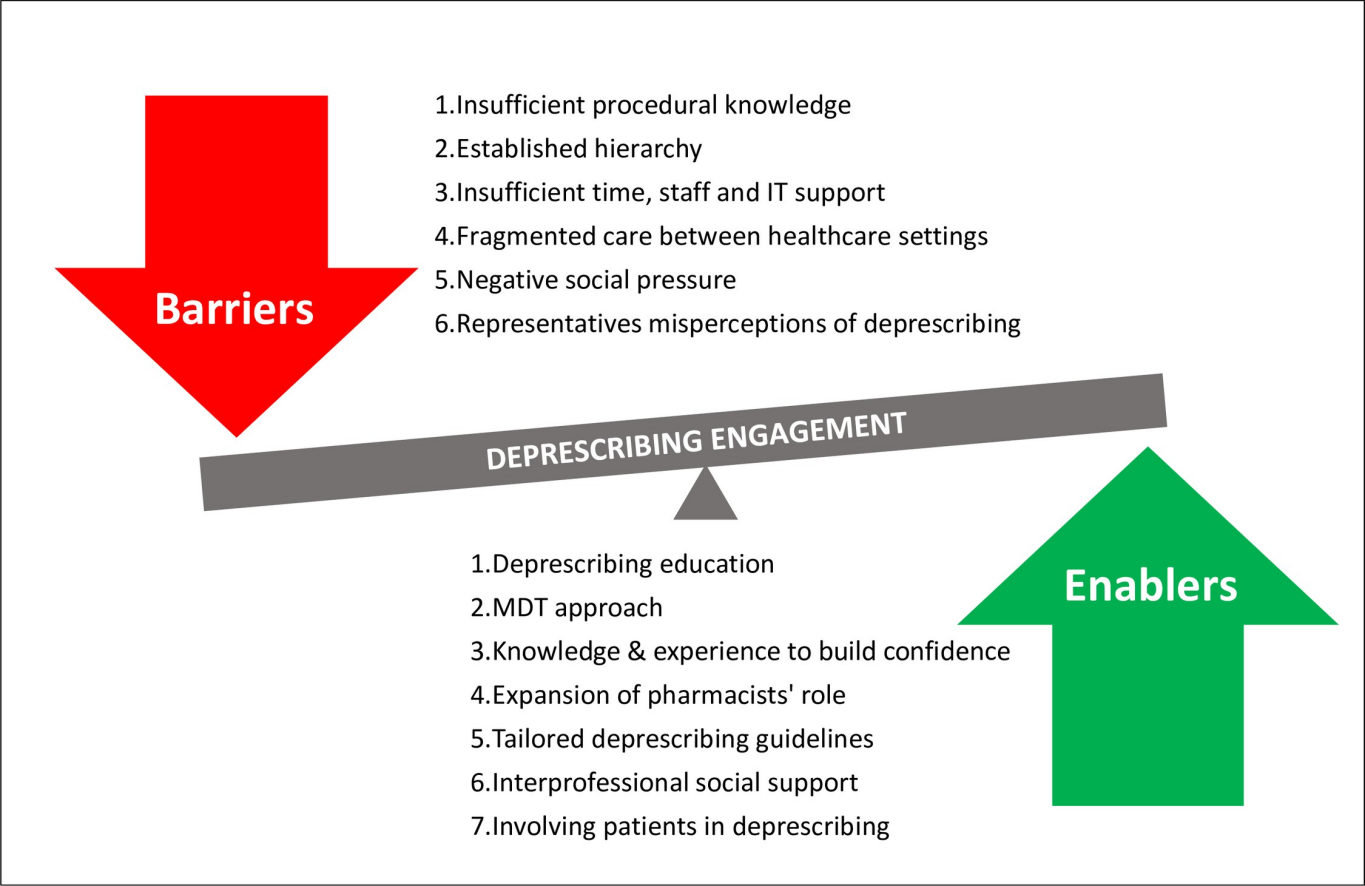
Dominant barriers, enablers and potential enablers affecting deprescribing engagement. (MDT = multidisciplinary team, IT = information technology).

Through inductive analysis, 38 themes related to barriers and enablers to deprescribing were generated. All themes mapped to the deprescribing framework. Dominant barriers and enablers mapped to five domains: ‘knowledge’, ‘social/professional role and identity’, ‘beliefs about capabilities’, ‘environmental context and resources’ and ‘social influence’ as identified in Table 2. Non-dominant barriers and enablers are summarised in Table 3. Constructs representing the major barriers and enablers identified are summarised in (Fig 1). The majority of the dominant domains provide evidence of both barriers and enablers.

**Table 2:** Dominant barriers (B), enablers (E), and potential enablers (PE) mapped to domains in the deprescribing framework.

Domain/construct	Description of barrier/enabler/potential enabler	Supporting quote	B/E/ PE
**Knowledge**			
Knowledge requirements	All HCPs from all settings required further education to incorporate deprescribing in their daily practice.	*“You need education… as in what each of their [HCPs] roles would be*, *what would be expected of them and then outline the benefits of that*.*”* Pharmacist #1 Private	B/E/ PE
Procedural knowledge	Practical application of deprescribing knowledge was not universal among HCPs.	*“Yeah*, *I would have come across the concept [of deprescribing] before… in college rather than in work*.*”* Pharmacist #3 Public	B
**Social/professional role and identity**			
Professional role; social identity; leadership; power; professional boundaries; *(fear)*	An ‘established hierarchy’ in long-term care was perceived by HCPs, with GPs having seniority in terms of responsibility for patient care. HCPs described fear and lack of authority to question the prescribing of GPs.	*“The difference in the superior and inferior*, *I mean the yeah*, *the position mainly*, *it’s just you have to follow the physician’s prescriptions or the advice*.*”* Nurse #2 Private	B
Professional role; social identity; Professional boundaries; *(interprofessional social support)*	GPs and pharmacists identified the benefits of expanding pharmacist’s scope of practice including during medication reviews and increasing their presence on-site, to provide clinical knowledge and support to the MDT.	*“Looking at the experience of other jurisdictions… there is an embedded pharmacist [in long-term care]*. *It is the pharmacist that is the person who has the technical knowledge oftentimes and can advise the GP what to do*.*”* GP #2 Public	PE
**Beliefs about capabilities**			
Beliefs; professional confidence; self-efficacy; perceived competence	Knowledge and experience gave HCPs confidence and enhanced their level of engagement with deprescribing in long-term care.	*“I’ve done the membership*, *I’ve 5 years hospital experience and a lot of GP years done as well so I think I’m kind of confident enough making decisions and backing up my decisions*.*”* GP #5 Private	E
**Environmental context and resources**			
Organisational alignment between long-term care and other settings	Insufficient communication and coordination between hospital and long-term care led to fragmented patient care, negatively affecting deprescribing.	*“There’s definitely… lack of crisp*, *fast*, *electronic communication between primary and tertiary care*. *It is just one of the biggest reasons there are drug errors and drug overprescribing*.*”* GP #6 Private	B
Resources: organisational; *(interprofessional social support)*	Face-to-face MDT meetings, conducted at regular intervals, between all three HCP groups facilitated deprescribing in long-term care.	*“There needs to be like a standard approach… making those quarterly reviews meaningful so that they’re not just to tick-box exercise… if it involved you know a sit-down MDT approach*.*”* Nurse #12 Private	E
Tailored material resources	Despite an awareness among a minority of HCPs of guidelines such as STOPP/START, most HCPs still called for clear tailored guidelines to provide confidence, support their decision making and help gain patient and representative buy-in.	*“One thing which I think would help is some tool… structured… where you would document in a very methodical fashion*, *why you are deprescribing a medication… And if that was a standard… and was based on whatever guidelines were available at the time*, *medicolegally defensible*, *I would feel much more confident stopping things*.*”* GP #7 Private	PE
Resources–Material	Insufficient resources negatively affected HCPs’ engagement with deprescribing. The major barrier was a perceived lack of time, followed by insufficient staff and lack of IT support.	*“[Deprescribing] can be quite time consuming… I think it should be a priority*, *but it may not always be due to time constraints*.*”* GP #3 Public	B
**Social influences**			
Interprofessional social support	Interprofessional social support and collaboration between all HCPs promoted engagement with deprescribing.	*“It would need to buy-in from all the parties… ’cause your prescriber does deprescribing*, *pharmacists implement the deprescribing… and the nurses observe the effect of deprescribing*. *But you very much have to have everybody on board*.*”* Pharmacist #6 Public	E
Intergroup conflict	Sometimes a misperception existed amongst family members that deprescribing means withdrawal of care; this negatively affected HCP’ engagement with deprescribing.	*“There is a misperception sometimes amongst family… that rationalizing medicine equals withdrawal of care as so it means that you’re actually taking away care from them*.*”* GP #6 Private	B
Patient social influence	For patients who are cognitively aware, involving them in deprescribing conversations can improve success rates.	*“With the patient*, *explain the pros and cons of each medication… I think they would be more on board with stopping a medication if they knew the risk benefit analysis of each*.*”* GP #3 Public	E
Social pressure	Social pressures to maintain the ‘status quo’ and not upset patients’ medication regimen negatively influenced HCP motivation to engage in deprescribing.	*“If it ain’t broke*, *don’t fix it… like it’s best practice at the moment*.*”* GP #7 Private	B

Domains and constructs from the ‘Best-fit’ framework of barriers and enablers to deprescribing in long-term care facilities by Heinrich and colleagues [39].

*(Italics)*: Related constructs from other domains. MDT = multidisciplinary team.

**Table 3:** Non-dominant barriers (B), enablers (E), and potential enablers (PE) mapped to domains in the deprescribing framework.

Domain/construct	Description of barrier/enabler/potential enabler	B/E/ PE
**Knowledge**		
Deprescribing knowledge	The majority of HCPs demonstrated some theoretical knowledge and awareness of deprescribing in long-term care.	E
**Skills**		
Skills; competence	The skills of all HCPs were identified as sufficient to support deprescribing.	E
**Social/professional role and identity**		
Professional role; leadership	Nurses were identified as organisational leaders, promoting deprescribing.	E
Organisational commitment: to long-term care	A perceived lack of commitment from private GPs to long-term care work was identified, by both GPs and other HCPs.	B
Organisational commitment: to deprescribing	HCPs described varied levels of commitment to deprescribing.	B/E
**Beliefs about consequences**		
Beliefs: deprescribing; outcome expectancies	Beliefs about deprescribing outcomes or previous experience of deprescribing influenced HCP engagement. Negative beliefs or experiences can negatively affect future deprescribing engagement. Similarly, positive beliefs or experiences can promote and encourage engagement.	B/E
Beliefs: about prescribing	HCPs’ beliefs about medications and prescribing can influence their engagement with deprescribing. For example, some GPs expressed concerns about prescribing specific classes of medication, while nurses discussed the importance of rationalizing medication regimens.	E
**Memory, attention and decision making**		
Decision making	Deprescribing decision-making involved a benefit-risk analysis, with focus on patient safety and wellbeing.	B
**Emotion**		
Fear	Fear of a negative patient outcome because of deprescribing, was a barrier for all HCPs.	B
**Reinforcement**		
Incentives	National cost savings for the health service, operational improvements and patient benefits were examples of rewards suggested by HCPs, which may support deprescribing engagement.	PE
	Incentives would support HCPs engaging with deprescribing in long-term care. Suggested incentives included financial and legislative requirements.	PE
**Goals**		
Goal priority	Deprescribing was not prioritised by HCPs for patients in long-term care.	B
**Environmental context and resources**		
Organisational culture and climate	The long-term care physical environment was thought by HCPs from both public and private settings, to be appropriate for deprescribing due to patient observations, perceived quality of nursing care and a controlled environment.	E
	The incorporation of deprescribing into existing quarterly medication reviews and integrating pharmacist services at the long-term care facility were suggested as mechanisms to increase HCP engagement with deprescribing.	PE
Resources: organisational; *(interprofessional social support)*	Healthmail, a secure email service between HCPs, facilitated deprescribing.	E
Tailored material resources	HCPs believed a structured procedure would be required to facilitate uniform engagement with deprescribing in long-term care facilities.	PE
**Social influences**		
Patient social influence	Patients, representatives’ expectations, and anticipated patient outcomes influenced HCP’ engagement with deprescribing.	B/E

Domains and constructs from the ‘Best-fit’ framework of barriers and enablers to deprescribing in long-term care facilities by Heinrich and colleagues [39].

*(Italics)*: Related constructs from other domains.

Analysis of domains across HCPs working in public and private settings identified different experiences related to three domains; ‘knowledge’, ‘reinforcement’ and ‘social/professional role and identity’ (Table 4). Commitment, both to the long-term care facility and deprescribing varied among HCPs in public and private settings. Staff from publicly-funded long-term care settings indicated a greater commitment to deprescribing than those working in the private sector. Similarly, HCPs’ opinions on guidelines differed, with HCPs in the private settings reporting limited awareness of deprescribing guidelines. HCPs working in the private sector referenced the need for financial incentives to support their engagement with deprescribing in long-term care to a greater extent than those in public settings.

**Table 4:** Analysis of public versus private opinions.

Domain	Quote	
	Public	Private
Guidelines: greater awareness of deprescribing guidelines among HCPs in public long-term care settings.	“Both in terms guiding my practice but also in terms of… audits I have done, STOPP/START and STOPPFrail are *the* guidelines.” *GP #2* “The STOPP/START guidelines are very useful for the elderly population, suggesting what should be stopped and when it should be stopped.” *Pharmacist #3*	“But we don’t have any guidelines as it is, that’s what I think (laughs)”. *Nurse #10* “Amm… so I suppose there’s… nothing really designed for… deprescribing you just rely on your usual resources, you know the BNF.” *GP #1*
Incentives: greater emphasis placed on the benefit of financial incentives to support deprescribing engagement from HCPs in private long-term care settings.	“I think that GPs would want to engage to be honest… I don’t think they would require more.” *GP #3* “I can’t see why you would need to incentivize it you know I mean it’s part of our job.” *Pharmacist #6*	“If GPs were remunerated for it [deprescribing], so if you had, you know, some protected time.” *GP #1* “I think pharmacists will… be going to the facilities… conducting these reviews which will be timely, and they want to be financially rewarded for that.” *Pharmacist #2*
Organisational commitment: HCPs in private long-term care settings showed limited commitment to long-term care work.	“It is good in fairness, if there’s any issues… we have a doctor team here four times a week.” *Nurse #5* “I’m the medical officer for a Community Nursing Unit. So we call there twice a week for ward rounds” *GP #4*	“The nursing home visits, there are not resourced, very often it’s our registrar… the least experienced of us, winds up doing the quarterly reviews.” *GP #5* “Every GP I think they’re coming quarterly to review their medicines and if they have any complaints, we can send the email.” *Nurse #9*
Deprescribing commitment: HCPs in private long-term care settings showed reduced engagement with deprescribing behaviours.	**“**I think on the whole… staff would engage with it [deprescribing].” *Nurse #4* “I think it’s something that definitely we could do from a daily basis.” *Nurse #7*	“I think that that’s why it’s [deprescribing] not routinely done at the moment because the stakeholders are all pulling in different directions.” *Pharm #2* “Prophylactic antibiotics are another [deprescribing target] say for UTIs… That can be difficult with the nursing staff sometimes to encourage. *GP #5*

Analysis of barriers and enablers specific to professional role, identified two findings specific to a healthcare profession; commitment and organisational leadership. Organisational commitment was a barrier attributed only to GPs. Nurses were identified as the organisational leaders of deprescribing by all HCPs based on their knowledge of residents and their role in communication with the GP which leads to medication review.

## Discussion

This qualitative study examined the perceived barriers and enablers to deprescribing among HCPs working in Irish long-term care facilities. To the best of our knowledge, this is the first study to utilise the TDF to analyse in-depth the factors which influence deprescribing behaviour in Irish long-term care facilities. Additionally, this study examines how these factors contrast across publicly-funded and privately-run long-term care settings. The major barriers to deprescribing included insufficient resources, lack of co-ordination between healthcare settings, negative social influences among colleagues and patient representatives’ understanding of deprescribing as a removal of care. The main enablers of deprescribing in long-term care were interprofessional support and patient involvement in deprescribing decision making. Potential enablers of deprescribing suggested by HCPs included deprescribing education, role expansion for pharmacists and tailored guidelines. These findings are important as they identify potential targets and important obstacles to consider, when designing a deprescribing intervention in long-term care.

Education was identified by all HCPs as a strong potential deprescribing enabler, similar to other deprescribing studies in this setting [24,42]. Educational opportunities include continuing professional development courses and on-site training, both of which have shown to effectively reduce PIM use in older adults [43]. HCPs proposed that a structured procedure, utilising a multidisciplinary team (MDT) and evidence-based tailored guidelines, would encourage deprescribing engagement. Wouters *et al* similarly suggest refining the STOPP/START guidelines for implementation in long-term care, to facilitate deprescribing decision making [44]. Considering the awareness among participants of STOPP/START, a future option could be the implementation of STOPPFrail, guidelines tailored for the frail older adult, often the population seen in long-term care [45].

Resources such as time, staff and documentation were described as insufficient in Irish long-term care facilities and a lack of communication and co-ordination between healthcare settings was believed to be responsible for the fragmented care for long-term care patients. These environmental barriers echo challenges identified in Irish primary care settings [46,47] and in international long-term care settings. [48–53]. Investment in resources, such as electronic health records could help to address these obstacles, supporting communication and co-ordination of patient care. Benefits of electronic health records in long-term care include improved access to patient information, increased documentation accuracy and better evidence-based practice implementation [54]. Furthermore, benefits of sharing health records between healthcare systems included improved communication and care planning, which could improve the time, administrative burdens and communication barriers which exist in long-term care [55].

Similarly, HCPs in this study reported insufficient financial resources to engage in deprescribing, which is a widely documented barrier in all healthcare settings [51,56–58]. Where financial incentives exist, HCPs are more positive toward deprescribing [33]. Currently in Ireland, HCPs are not reimbursed to provide deprescribing services in long-term care facilities. Furthermore, community pharmacists are reimbursed per item dispensed and thus, deprescribing could be perceived as a potential financial disincentive. A similar challenge exists in Germany, with pharmacists explaining that by deprescribing PIMs, they are “doing the right thing, and getting less for it” [59]. This remains a barrier which requires change in the reimbursement structure and potential governmental incentivisation to overcome. Despite lack of resources and communication channels, the long-term care environment was regarded by participants as an ideal setting for deprescribing. This is due to the increased patient observations compared to the community setting, perceived quality of nursing care and a controlled environment.

Deprescribing in long-term care is negatively impacted by the widespread perception of an ‘established hierarchy’, where GPs are regarded as having seniority above pharmacists and nurses [57,60]. Interestingly, in this study, GPs did not feel pressure to conform to the prescribing practices of consultants, as they perceive themselves to be the HCP responsible for patient care. This contrasts with the international opinion where consultants occupy the apex of the hierarchy, despite little difference in consultant roles between jurisdictions [48,50,51,61]. Future deprescribing interventions could benefit from incorporating the entire MDT to mitigate fears of perceived hierarchy. Reinforcing this, HCPs called for an expansion of integrated pharmacist services in long-term care. Pharmacists are identified as deprescribing facilitators, offering knowledge, support and confidence in decision making. Similar campaigns exist internationally promoting the integration of pharmacists into long-term care, to provide clinical services, participate in discussions and educate staff [62]. A systematic review by Sadowski *et al* concluded that there is evidence to support the integration of a pharmacist into long-term care, particularly to provide medication reviews to increase medication appropriateness, supporting the response from study participants [63].

Comparing the opinions of HCPs from public and private settings, HCPs working in privately-funded long-term care facilities had a limited awareness of deprescribing guidelines and increasingly called for financial incentives compared to their counterparts in publicly-funded settings. GPs and pharmacists from both settings are remunerated for care provided to patients; however, HCPs felt that the current format and extent of reimbursement for private long-term care work isn’t sufficient to warrant the time required to engage with deprescribing. This is an important consideration, if developing a deprescribing intervention for long-term care. Similarly, commitment levels to long-term care work varied among HCPs. GPs and pharmacists in public settings are “contractually required” to attend the long-term care setting. Contrastingly, in the private sector, GPs explained that often it is their registrar who attends the long-term care facility, as they themselves do not have sufficient time. This perceived lack of interest and commitment appears to be an ongoing problem within Irish healthcare, as it was highlighted in 2015 by Nursing Homes Ireland as an issue of concern [64]. Bolmsjö *et al* similarly identified contrasting interest in long-term care work among HCPs from two jurisdictions with different organisational structures [33]. A UK study which switched the frequency of long-term care visits from *ad hoc* to weekly, resulted in reduced hospital admissions and care home visits. The routine structure allowed ‘ward round style’ care, similar to public long-term care facilities, supporting HCP education and decision making [65]. Incorporating routine visits into the Irish private long-term care sector could support deprescribing engagement; however, reimbursement may be required to incentivise GPs to dedicate time to such work. Alternatively, an organisational switch toward a medical officer model could be considered, creating a defined, reimbursed role.

The foundation to support deprescribing exists in long-term care. The medication review is identified, both from this study and the wider literature, as an ideal opportunity to engage with successful deprescribing [24,66,67]. However, in the Irish context this needs reform, switching from a “tick-box” exercise to a meaningful intervention, facilitating deprescribing. Suggested changes include creating a structured procedure, incorporating the MDT, shared decision-making and tailored guidelines, to increase uniformity, acceptability, and engagement.

### Strengths/Limitations

Strengths of this study include the chosen methodology and sampling strategy. Qualitative description allowed for a rich description of experiences, from the perspective of participants [68]. Purposively sampling HCPs from both private and public long-term care settings, in addition to triangulation of opinions from multiple HCPs involved in the immediate care of long-term care residents, offers an in-depth understanding of the barriers and enablers present in different types of long-term care settings operating in Ireland. The results will inform the development of an intervention that can be tailored to each setting, but also providing contextual information to support transferability of the findings. This study mapped barriers and enablers to a TDF-informed deprescribing framework, increasing the utility of the study to inform intervention development [69]. Using a deprescribing framework, created to identify barriers and enablers in long-term care, allows incorporation of contextual influences which can moderate behaviour. Such motives may not be captured in a purely TDF-focused deductive analysis [70].

Some limitations of this study exist. The interviewer is a pharmacist. This was known to the participants at the time of the interview and may have affected responses; participants tailoring their opinions to meet anticipated expectations. However, conversations were meaningful and engaging. This also could have introduced professional bias during analysis, as the majority of the research team were pharmacists. Long-term care facilities were asked to introduce the project to their staff, therefore the risk of selection bias cannot be ruled out. Due to COVID-19 restrictions, interviews were conducted via Microsoft Teams ©. The use of technology may have deterred potential participants from engaging in the research, possibly due to technical issues, planning, privacy and rapport [71].

## Conclusion

HCPs working in Irish long-term care understand deprescribing; however, it is not routine practice. Deprescribing interventions can build on existing structures, such as the quarterly medication review by incorporating a MDT approach, drawing on the knowledge and experiences of all HCP in a structured, routine manner. A deprescribing algorithm, supported with tailored guidelines and education could support engagement from all HCPs, both during formal medication reviews and for any change in a patient’s clinical condition, requiring deprescribing. Furthering knowledge and encouraging engagement for all HCPs can offer the confidence required to make evidence-based deprescribing decisions in long-term care. It is suggested that the environment of the long-term care facility is an ideal setting for deprescribing, however limited resources in addition to the poor co-ordination with other healthcare settings, confines HCPs from successfully deprescribing. Expanding pharmacists’ role to include deprescribing responsibilities may help to overcome time constraints for other HCPs. Patient engagement in the form of shared decision making is an option to overcome the anticipated negative response from patients and representatives toward deprescribing. HCPs consider it feasible to implement deprescribing in long-term care, however an intervention must be designed to account for the nuanced barriers which exist in both settings.

## Supporting information

S1 File (DOCX)
