# Supplementary material for: Barriers and enablers to deprescribing in long-term care: A qualitative investigation into the opinions of healthcare professionals in Ireland
Source: PLoS One. 2022 Dec 15;17(12):e0274552. doi: 10.1371/journal.pone.0274552 (PMC9754218; doi:10.1371/journal.pone.0274552)
Supplement: S1 File — (DOCX) [file pone.0274552.s001.docx]

Supplemental Information 1: COREQ-32

| **Domain 1: Research team and reflexivity** |  |
| --- | --- |
| *Personal characteristics* |  |
| Interviewer | Clara H. Heinrich |
| Credentials | BPharm, MPharm, MPSI |
| Occupation | Pharmacist. |
| Gender | Female |
| Experience and training | Qualitative research training at university level. |
| *Relationship with participants* |  |
| Relationship established | A small selection of HCP had a previous professional relationship with the interviewer through her role as a pharmacist, prior to study commencement. |
| Participant knowledge of the interviewer | Participants were aware of the researcher’s profession and aims for the research of developing a deprescribing intervention. |
| Interviewer characteristics | No characteristics were reported. |
| **Domain 2: Study design** |  |
| *Theoretical Framework* |  |
| Methodological orientation and theory | Qualitative description methodology using thematic analysis to develop themes and mapping to TDF-informed framework of barriers and enablers |
| *Participant selection* |  |
| Sampling | Convenience and stratified purposive sampling. |
| Method of approach | Phone call and/or email. |
| Sample size | Between 6-10 participants per HCP group. |
| Non-participation | No participants dropped out. |
| *Setting* |  |
| Setting of data collection | Microsoft Teams. |
| Presence of non-participants | N/A |
| Description of sample | Table 1 |
| *Data collection* |  |
| Interview guide | Semi-structured interview guide piloted on one GP, pharmacist, and nurse. |
| Repeat interviews | No. |
| Audio/visual recording | Either, depending on participant preference |
| Field notes | No |
| Duration | Average: 24 mins (9 mins - 65min) |
| Data saturation | No, sample size identified via information power. |
| Transcripts returned | No |
| **Domain 3: Analysis and findings** |  |
| Number of data coders | Inductive coding conducted by one researcher, mapping of inductive codes to ‘best-fit’ framework conducted by two researchers. |
| Description of the coding tree | Initially inductive coding, then mapped to ‘best-fit’ framework of deprescribing barriers and enablers. |
| Derivation of themes | Derived from the data, informed by researchers’ knowledge and experience. |
| Software | NVivo |
| Participant checking | No. |
| *Reporting* |  |
| Quotations presented | Yes. |
| Data and findings consistent | Yes. |
| Clarity of major themes | Yes. |
| Clarity of minor themes | Yes. |
